# Developing Angelman syndrome-specific clinician-reported and caregiver-reported measures to support holistic, patient-centered drug development

**DOI:** 10.1186/s13023-023-02729-y

**Published:** 2023-06-22

**Authors:** Siobhan Connor-Ahmad, Jorrit Tjeertes, Michael Chladek, Louise Newton, Tara Symonds, Susanne Clinch, Brenda Vincenzi, Fiona McDougall

**Affiliations:** 1 Roche Products Ltd., Welwyn Garden City, AL7 1TW UK; 2 F. Hoffmann-La Roche Ltd., Basel, Switzerland; 3 Clinical Outcomes Solutions, LLC, Chicago, USA; 4 Clinical Outcomes Solutions Ltd., Folkestone, UK; 5 Genentech Inc., San Francisco, USA

**Keywords:** Angelman syndrome, Caregivers, Children, Clinical outcome assessment

## Abstract

**Background:**

Angelman syndrome (AS) is a rare, heterogenous neurogenetic condition, which significantly impacts the lives of people with AS and their families. Valid and reliable measures reporting key symptoms and functional impairments of AS are required to support development of patient-centered therapies. We describe the development of clinician- and caregiver-reported, AS-specific Global Impression scales for incorporation into clinical trials. Best practice US Food and Drug Administration guidance for measure development was followed with input from expert clinicians, patient advocates, and caregivers during content generation and refinement.

**Results:**

Initial measurement domains for the Symptoms of AS—Clinician Global Impression (SAS-CGI) and the Caregiver-reported AS Scale (CASS) were identified from a conceptual disease model of AS symptoms and impacts, derived from interviews with caregivers and clinicians. Two rounds of cognitive debriefing (CD) interviews were performed; clinicians debriefed the SAS-CGI, with patient advocates and caregivers debriefing the CASS to ensure relevance and comprehension. Feedback was used to refine items and ensure wording was age-appropriate and captured AS-specific symptoms, as well as associated impacts and functional impairments. The SAS-CGI and CASS capture global assessments of seizures, sleep, maladaptive behaviors, expressive communication, fine and gross motor skills, cognition, and self-care, which were determined by clinicians, patient advocates, and caregivers to be the most challenging aspects of AS. Additionally, the measures include items for assessing overall AS symptoms and the meaningfulness of any change. In addition to ratings for severity, impact, and change, a notes field was included in the SAS-CGI to provide the rationale for the chosen rating. CD interviews confirmed the measures covered key concepts of AS from the perspective of clinicians and caregivers, and demonstrated that the measures’ instructions, items, and response options were clear and appropriate. Interview feedback informed adjustments to the wording of the instructions and the items.

**Conclusions:**

The SAS-CGI and CASS were designed to capture multiple AS symptoms, reflecting the heterogeneity and complexity of AS in children 1 to 12 years old. These clinical outcome assessments have been incorporated into AS clinical studies, which will allow for the evaluation of their psychometric properties and inform further refinements if needed.

## Background

Angelman syndrome (AS) is a rare neurogenetic condition [1], with a prevalence of approximately 1 in 22,000 births, caused by the loss of function of the maternally inherited ubiquitin-protein ligase E3A gene on Chromosome 15 [2–4]. Presentation of AS symptoms and impairments occur early in life with developmental, behavioral, and medical challenges, that vary and change with age, and can have a significant impact on both individuals and their families [5]. Conceptual disease models of AS have been developed to identify the symptoms and functional impairments that have important impacts on people with AS and their families [6, 7]. Identified AS-defining domains include seizures, sleep disturbance, maladaptive behaviors, impaired expressive communication, poor fine motor skills, poor gross motor skills, impaired cognition, and limited self-care abilities [7]. People with AS usually have a lifespan comparable to the general population; however, during their life they require continuous care and cannot live independently as adults [1, 8].

Current interventions for AS comprise of pharmacologic treatments, which can include sleep and seizure medications, as well as supportive therapies such as physical and speech therapy [1, 9]. Clinical outcome assessments (COAs) evaluating the key symptoms and functional impairments of AS are important to support therapy development and the accurate assessment of changes in AS symptoms [7, 10]. The US Food and Drug Administration (FDA) guidelines have highlighted the importance of patient-centered drug development and outline a standard process to ensure COAs used in clinical trials are underpinned by an understanding of the most relevant symptoms, plus are valid, reliable, and able to detect change [11, 12]. At the time of this study, no AS-specific COAs that holistically reviewed the status of patients with AS were publicly available. Hence, there was a need to develop measures that capture the complexity and heterogeneity of AS symptom burden from the clinician and the caregiver perspectives.

An approach that has been successfully used in other heterogenous conditions is the use of syndrome-specific Clinician Global Impression (CGI) scales [13–15]. These were introduced by Guy, et al. and have since been adapted to come in the form of a CGI – Severity (CGI-S) scale [16]. This allows clinicians to rate the severity of symptoms, as well as CGI – Change (CGI-C) and CGI – Improvement scales, which allow clinicians to rate any changes in symptoms [15, 17].

It is important to capture the perspectives of both clinicians and caregivers to understand the impact that AS has on individuals living with this condition. Caregivers provide insights into the impact of AS symptoms and impairments on day-to-day life, while clinicians can contextualize the symptom severity in relation to the broader AS population. This dual perspective is especially important since people with AS have impaired communication and cognition, which prevents them completing patient Global Impression scales or participating in concept elicitation interviews to aid in the development of similar COAs [7, 8].

Here, we describe the robust development of clinician- and caregiver-reported, AS-specific Global Impression measures that capture severity, impact, and change across multiple AS symptoms and for overall AS in children 1 to 12 years of age with AS.

## Results

### Participant demographics

**Fig. 1:**
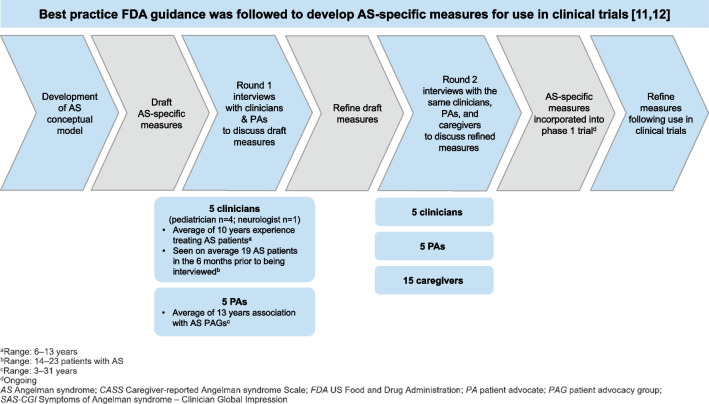
Development process of the SAS-CGI—Severity and—Change and the CASS—Impact and—Change

Five clinicians with both expertise in AS plus a Doctor of Medicine (MD) degree were interviewed to inform the content of the SAS-CGI measures (Fig. 1). To inform the content of the caregiver measures, interviews were performed with five patient advocates (PAs) from different AS-specific patient advocacy groups (PAGs), with a mean of 13 years (range: 3–31 years) of affiliation, plus 15 caregivers who were a parent to a child with AS (Fig. 1, Table 1). The 15 caregivers supported a total of 15 children with AS, with an age range of 1–12 years (Table 2).

**Table 1:** Caregiver demographics

Demographic variables	Total (N = 15)
*Caregiver’s age, years*	
Mean (SD)	38.6 (5.4)
Median	39.0
Range	27.0–50.0
*Caregiver’s sex, n (%)*	
Female	14 (93.3)
Male	1 (6.7)
*Caregiver’s ethnicity, n (%)*	
Hispanic/Latino	1 (6.7)
Not Hispanic/Latino	14 (93.3)
*Caregiver’s race, n (%)*	
White/Caucasian	13 (86.7)
Black/African American	1 (6.7)
Asian/Asian American	1 (6.7)
*Caregiver’s highest level of education, n (%)*	
Some college or certification program	4 (26.7)
College, technical college, or university degree (2- or 4-year)	7 (46.7)
Graduate degree (MS, PhD, MD, etc.)	4 (26.7)
*Caregiver’s work status, n (%)*	
Employed/self-employed full time (≥ 40 h per week)	7 (46.7)
Employed/self-employed part time (< 40 h per week)	2 (13.3)
Homemaker	5 (33.3)
Unemployed	1 (6.7)

*MD* Doctor of Medicine; *MS* Masters of Science; *PhD* Doctor of Philosophy; *SD* standard deviation

**Table 2:** Child demographics and clinical characteristics

Demographic variables	Total (N = 15)
*Child’s age, years*	
Mean (SD)	5.3 (3.8)
Median	4.0
Range	1.0–12.0
*Child’s sex, n (%)*	
Female	5 (33.3)
Male	10 (66.7)
*Child’s ethnicity, n (%)*	
Hispanic/Latino	3 (20.0)
Not Hispanic/Latino	12 (80.0)
*Child’s race, n (%)*	
White/Caucasian	12 (80.0)
Black/African American	1 (6.7)
Asian/Asian American	1 (6.7)
Other^a^	1 (6.7)
*Time since diagnosis, months*	
Mean (SD)	47.1 (43.0)
Median	28.5
Range	2.0–124.0
*Genetic subtype of AS, n (%)*	
Deletion	10 (66.7)
Mutation	2 (13.3)
Imprinting defect	2 (13.3)
Paternal uniparental disomy (UPD)	1 (6.7)
*Caregiver-reported severity over past 3 months, n (%)*	
Very mild	4 (26.7)
Moderate	5 (33.3)
Severe	5 (33.3)
Very severe	1 (6.7)

^a^Child was identified by the caregiver as being multiracial

*AS* Angelman syndrome*; SD* standard deviation; *UPD* uniparental disomy

### SAS-CGI measure development

#### Round 1 interviews

During open-ended discussions, clinicians confirmed, either spontaneously or through probing, that the following symptoms included in the SAS-CGI-S and SAS-CGI-C were of key importance: seizures, sleep disturbance, maladaptive behaviors, impaired expressive communication, poor fine motor skills, poor gross motor skills, impaired cognition, and limited self-care abilities (Table 3).

**Table 3:** Clinician and patient advocate confirmation of key symptom relevance

Symptom	Interviewees	Spontaneous (n)	Probed (n)	Total (n)	Quote from clinician interviews	Quote from PA interviews
Seizures	Clinicians	4/5	1/5	5/5	“*Um, seizures are very important 'cause there is a difference of a priority from having a seizure and being safe and having it… you know, putting a person in jeopardy versus, um, say, uh, not being able to, um, walk correctly but use an aid.*”	*“Yeah, mo- most of the symptoms are gonna be seizures and, and sleep.”*
	PAs	5/5	0/5	5/5		
Sleep	Clinicians	2/5	3/5	5/5	*“Yeah, difficulty sleeping, uh, limited in their duration of sleep. If they have, yes, that's an often a common issue.”*	*“The sleep can be really hard, because if they aren't sleeping at night, they'll often fall asleep during the day. And then, they can't have a normal life, because they have to sleep during the day, and they have to miss out on […] school, or activities, or different things.”*
	PAs	5/5	0/5	5/5		
Nonverbal communication	Clinicians	2/5	3/5	5/5	*“Some of them will be nonverbal, you know, but will go on to learn, uh, very effectively at communicating sign language. And others will overcome, you know, that and it really just depends on the severity at that point.”*	*“The communication part is very big because um, unless they- they're… are given outside ways to do that using a communication device or something, they have no way to indi- indicate pain, tell you what happened when they weren't in front of your eyes, um, tell you what's bothering them when they have anxiety and um, the fine motor problems and the hypermoticity really um, limit the amount of sign language that Angelman people can do well.”*
	PAs	3/5	2/5	5/5		
Verbal communication	Clinicians	5/5	0/5	5/5	*“There's so many things, it [developmental delays] could be from, uh, hand gestures, and their verbal and nonverbal communications, depending on how young they are […] whether they mumble or try to speak, and, it's, I mean, a lot of factors.”*	*“There are some kids out there that are, are able to say a few words, um, very basic words, but a few words or you know, a, a different way of communicating that is not verbal. So I mean the hope is that there is, you know, that there can be some verbal words that are used, used, but if not that there is some sort of forms, um, or outlets to allow them to communicate.”*
	PAs	2/5	3/5	5/5		
Fine motor	Clinicians	2/5	3/5	5/5	*“They have problems with motor skill- skills, especially fine motor skills, and sometimes gross motor skills, th- those, and, and, they're just, they have got developmental problems, uh, which starts right around, uh, really noticeable from 6 months on, I’d say, and especially getting closer to being a toddler.”*	*“Yes, fine, fine motor skills is, is, uh, absolutely up there as far as, um, uh, a symptom of, of Angelman syndrome. […] Be it taking a block and putting on top, top of another one, or going to a… I’m thinking of everyday life, going to a door and turning the handle to open the door. It's difficult for, for Angelman, uh, uh, people to do that.”*
	PAs	1/5	4/5	5/5		
Gross motor	Clinicians	5/5	0/5	5/5	*“Basically it’s just about crawling, um, walking, um, able to hold balance, basically, delays that a child with difficulty can start, you know, crawling, walking, running, where this child may have complete delays.”*	*“Um, a lot of the kids start walking by the age of 4, um, but they still can have difficulty kind of walking smoothly and running properly or, um, balancing, you know, when there's a curbing or steps, so gross motor skills in the school-age kids are not as worrisome as the fine motor, really. But I mean, it still is a problem, and then as the kids get older, after the age of 12, their gross motor skills get worse. So in the age that you spoke of, I think the gross motor skills are, are definitely important, uh, but maybe not quite as important as some of the other things.”*
	PAs	2/5	3/5	5/5		
Cognition	Clinicians	1/5	4/5	5/5	*“Yes. Significant cognitive the, uh, uh, abilities they tend to. And that persists even as they get older.”*	*“Um, that's, I mean, if you look at the, the population most, uh, Angelman is, is a deletion and, and their cognitive ability to, you know, really to learn is, is poor. Um, learning colors or, or symbols and, and matching them. They might be able to do it one week and then not the next week.”*
	PAs	3/5	2/5	5/5		
Maladaptive behaviors	Clinicians	2/5	3/5	5/5	*“It's just as they get a little older we look at their behaviors as far as, uh, what's appropriate, uh, behavioral patterns for their age-appropriateness. Uh, we notice, uh, possibly hyperactivity, uh, whether they're clapping their hands, whether they're laughing and over ha- overly happy, or in, we notice if some of their, uh, some of their be- um, behaviors are overly repetitive. You know, you know, and depending on how old they are, up until 12, we notice that sometimes they refuse to, uh, engage or particip- uh, participate […] in activities.”*	*“Um, the biggest struggles I see are seizures and behavior. […] Um, the aggression, hair pulling, pinching, kicking, biting. Those kinda things.”*
	PAs	3/5	2/5	5/5		
Caring for self	Clinicians	1/5	4/5	5/5	*“Some will get to the point where they can easily, you know, dress themselves, they might struggle with fine motor skills, buttoning things, zipping things, um, where others will have to have complete assistance.”*	*“And then also just daily living skills, like brushing teeth, eating, buttoning, um, buttons, um, m- manipulating, I mean using a pencil or a pen. So most of the kids really have difficulty with all of those things.”*
	PAs	3/4^a^	1/4^a^	4/4^a^		

^a^One PA did not spontaneously mention and was not probed about caring for oneself independently as a core symptom

*PA* patient advocate

**Fig. 2:**
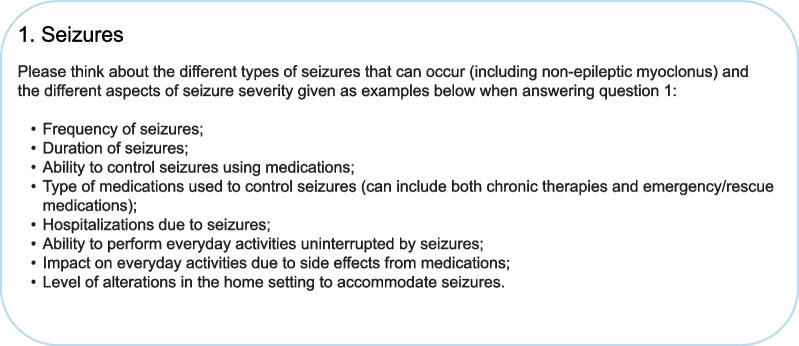
Example rating guidance

When assessing the severity of symptoms of children with AS, two out of four clinicians asked reported using other children with AS as a reference population, while the other two clinicians made comparisons to neurotypical children. Two clinicians agreed that it would be relevant to consider chronologic age and development expectations in the SAS-CGI-S. As a result, a rating guidance and scale manual was introduced to support consistent rating for each item across different clinicians, including in relation to the reference population. The guidance also provides greater specificity and direction to clinicians regarding the aspects of a domain to consider when responding (Fig. 2).

When asked about the range of response options in the SAS-CGI-S, one clinician out of the two explicitly asked reported that they were adequate, while the other suggested that the “very mild” and “very severe” options may be redundant. The description or examples for each response option of the SAS-CGI-S, and tables describing each response option of the SAS-CGI-C were removed based on feedback from a clinician: “*You don't want something that is complex or lengthy. This needs to be a very quick reaction answer and move forward.*” With respect to the SAS-CGI-C, both clinicians who were asked indicated that the level of change response options appropriately covered the range of possible responses.

Item-level feedback on the domains was also provided. For example, regarding seizures, two of the three clinicians debriefed on the seizure item of the SAS-CGI-S reported that frequency of seizures was of particular importance when determining seizure severity: *“So then you have the number and then we can move into severity. If they're having one seizure once a year or they're having 12 seizures a month, we know there's more of a problem.”* The other clinician noted that the ability to control seizures with medication was relevant to assessing seizure severity: *“There's a whole slew of different medications you could put a child on and seeing how they respond…, the response from maybe they're having mild seizures, and now it's completely controlled, to maybe severe, and now it's mild.”*

With regard to the expressive communication domain, all three clinicians debriefed on the nonverbal communication item reported that the list of communication approaches provided were helpful and relevant**.** Both clinicians debriefed on verbal communication understood verbal communication to include use of vocabulary words and not just vocalizing sounds: *“Yes, babbling is nonverbal. I’m talking words, words actually words. Spelled out words.”*

Following these first round interviews, the SAS-CGI measures were modified. Any changes made to the wording of the SAS-CGI-S items were applied to the SAS-CGI-C items, where appropriate. Item-specific wording was updated as appropriate. For example, in the seizure item, the consideration of “level of alterations to caregiver’s daily lives and the home setting to accommodate seizures” was changed to remove “to caregiver’s daily lives” to focus on the patient rather than the caregiver. The “not relevant” response option in the SAS-CGI-S scale was also updated to “none” following feedback from two clinicians who reported the need for a response option to indicate that the symptom was not being experienced at all. This could include when describing seizures or a symptom that had not yet developed at that individuals’ age, such as walking for young children. Verbal and nonverbal items were combined into expressive communication because it was determined that the overall aim of the item was to measure expressive communication difficulties, not the mode of expressive communication.

#### Round 2 interviews

A second round of interviews with the same clinicians was conducted to obtain feedback on the revised SAS-CGI measures. During open-ended discussions, all the clinicians who were asked to describe the instructions in their own words were able to do this in a way that aligned with the intended meaning, including: the rating guidance (5/5), the SAS-CGI-S (5/5), and the SAS-CGI-C (3/5): “*Those instructions [in the SAS-CGI-S] seem to be pretty unambiguous.*” None of the clinicians had any suggested changes to the measures’ instructions. One clinician suggested that the meaning of “none” should be clarified in the rating guidance. As a result, the SAS-CGI-S measures were modified to add additional explanation on when a clinician should select “none” for each domain. Four of the five clinicians expressed concern about assessing severity of developmental delays in very young patients aged 12–18 months. Four of the five clinicians reported they would use the notes section to explain the reasoning behind their response choice as was intended.

Item-level feedback on the domains was also provided. For example, regarding the seizures domain, four of the five clinicians agreed that the rating guidance covered the key aspects of seizures to consider: *“I mean… what you're using is that baseline to get their [seizure severity]. You know, frequency, duration, control, the efficacy of the medication… Were there any hospitalizations?… Are their days interrupted?… What alterations have to be made in the home?… That's very important, because those are the main things that would come up for any clinicians.”* Two clinicians suggested considering seizure history, types of medication used, and side effects of anti-seizure medications.

With regard to the expressive communication domain, all four clinicians who were asked confirmed that it made sense to combine the items on verbal and nonverbal expressive communication “*because it’s quite similar [in terms] of how you’re analyzing it,”*.

All three clinicians who were asked indicated that the overall AS item was relevant and interpreted it as asking them to consider their rating for all previous items. Additionally, four of the clinicians who were asked, indicated that the meaningfulness of change item was relevant. Following the Round 2 interviews with clinicians, the meaningful change item of the SAS-CGI-C, the final response option of “Not relevant—no overall change in their AS severity” was rephrased to “Not applicable—no change in their overall AS severity.” Additional considerations were added to the rating guidance.

### CASS measure development

#### Round 1 interviews

During open-ended discussions, all PAs confirmed that the symptoms included in the CASS measures were of key importance for individuals with AS (Table 3). When PAs were asked about the reference population they expected caregivers would consider when choosing a response option for their child with AS, two out of four PAs who were asked reported that caregivers would consider their child compared with others with AS, while the other two reported that the caregivers’ reference population would depend on the age of their child. PAs also noted that it could be important to specify a reference population within the instructions of the measures to clarify how caregivers should rate their child. This feedback from PAs highlighted the need to probe further on the reference population caregivers use when answering the CASS-I measure.

Item-level feedback was also provided. For example, one PA reported that the meaningful change item of the CASS-C would be relevant and understood by caregivers. In relation to the cognition items, two PAs felt when answering the item, caregivers would think about their child’s ability to focus attention or follow directions.

Following the Round 1 interviews with PAs, the CASS measures were modified to ask about the impact of symptoms instead of severity. This is because it became clear based on the discussion from Round 1 interviews that caregivers were best and uniquely placed to assess the impact of AS on their child’s daily life, rather than report symptom severity relative to other children.

**Fig. 3:**
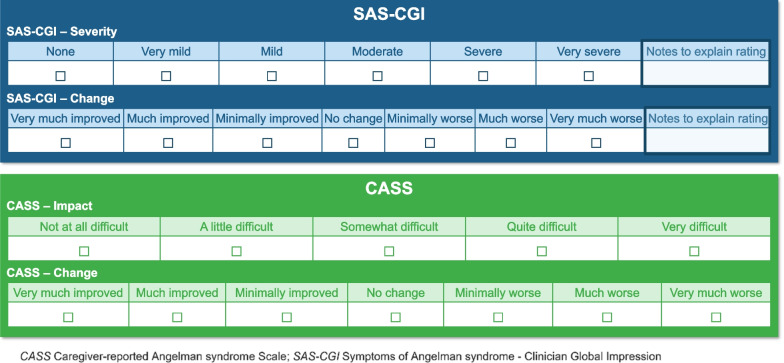
Response options for the SAS-CGI—Severity and—Change and the CASS—Impact and—Change measures

Additional updates included simplifying the response options of both CASS measures to remove detailed descriptions for each response option since the measure changed from asking about severity, to impact. Instead, for the CASS-I caregivers were asked to rate the level of difficulty the child had in relation to the AS symptom over the past 7 days using a 5-point response scale. For the CASS-C, caregivers were asked to rate the amount of change in the AS symptom or in overall AS compared with the start of the clinical study using a 7-point response scale (Fig. 3). The “Not relevant” response option was removed from the CASS-C since the “No change” option could be selected when the child had no history of a symptom.

#### Round 2 interviews

The same five PAs reconfirmed during Round 2 interviews that the CASS measures covered the key domains of AS that are important to caregivers. The four PAs who were asked confirmed that the response options were suitable for CASS-I, and all five PAs confirmed suitability for CASS-C. However, one PA suggested it was unlikely a caregiver would select anything other than “very impacted” for the overall AS item of the CASS-I**.** To keep response options consistent across items, no changes were made to this item. Three of the five PAs reported that the items would need to be revised for children aged < 1 year or if the measure were to be used outside of its intended context of use (i.e., children 1 to 12 years of age with AS).

All 15 caregivers debriefed on the revised CASS measures confirmed that the domains were relevant and of key importance to caregivers of children with AS. As one caregiver noted, *“You've hit the big ones. You know, self-care, […] fine motor skills, speech of course is a biggy, seizures is the second biggy.”* All caregivers who were asked demonstrated good understanding of the measures’ instructions and most caregivers agreed that the response options were suitable for each item.

Overall, eight out of 15 caregivers reported thinking only of their child without consideration to other children either with or without AS, *“I was trying to think of it as what her normal is, not a typical child, not another Angelman child, but what is her normal because I would need to know if something is different over 7 days for her.”* Other comparisons included: considering their child relative to other children both with AS and without (3/15), comparing their child solely with other children with AS (1/15), considering their child solely compared with children without AS (1/15), and considering them relative to both other children without AS and their own child over time (1/15). Two of the PAs suggested that specifying a comparison group in the instructions of the CASS-I could be helpful for caregivers. One PA similarly suggested clarifying for each item that the caregiver should answer *“compared to a person their own age.”* As a result, specification was added in the CASS-I instructions for caregivers to consider their child compared with children without AS who are approximately the same age as their child when choosing their response.

Feedback from caregivers at the item level showed most items were understood. However, some refinements were suggested to improve understanding. For example, while the expressive communication item was understood by 10 of the 15 caregivers, five caregivers were not sure what they should consider when answering the question, namely if they should consider how impacted their child’s communication was with themselves as caregivers or with other people more generally, who may have a harder time understanding the subtlety of the child’s style of communication. As a result, wording was added to the communication item to specify “communicating with people outside of their close family.” Additionally, extracted response data showed that caregivers often responded to the seizure item with “not at all impacted”, despite indicating that their child had been given, or was currently using seizure medication. The seizure item was therefore adjusted, adding different aspects of seizures and their impacts that caregivers could consider when answering, including medication.

**Fig. 4:**
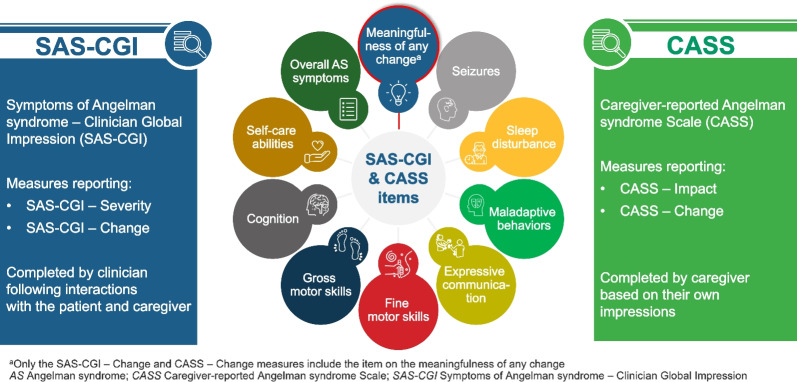
Overview of SAS-CGI—Severity and—Change measures and the CASS—Impact and—Change measures

Following the iterative process of CD and refinement, the domains and response options included in the final SAS-CGI and CASS measures are shown in Figs. 3 and 4.

## Discussion

This manuscript describes the development of AS-specific, clinician- and caregiver-reported Global Impression measures, the SAS-CGI and CASS, for children 1 to 12 years old with AS. These measures capture the key AS symptoms, both independently and holistically via an overall AS item. These measures were developed through multiple rounds of qualitative interviews that elicited feedback from expert clinicians, PAs, and caregivers to children with AS.

Clinicians, PAs, and caregivers confirmed that the eight key domains represented the most impactful symptoms and functional impairments for individuals with AS, namely, seizures, sleep disturbance, maladaptive behaviors, impaired expressive communication, poor fine motor skills, poor gross motor skills, impaired cognition, and limited self-care abilities. These domains were incorporated into the measures to ensure that the most challenging aspects of AS were captured, which is in line with the FDA guidance for patient-centered drug development [11, 12]. Gross and fine motor skills were separated due to the different impacts on daily living activities, specifically difficulties crawling or walking versus difficulties grasping or manipulating an object. Both the SAS-CGI and CASS measures include a single item for each domain along with an additional single item relating to overall AS symptoms. Furthermore, to support the identification of meaningful change, a specific item to capture this was included in both SAS-CGI-C and CASS-C measures.

Given the heterogeneity of AS and limited COAs available, modified Global Impression items were deemed appropriate to assess symptom severity or impact and how symptoms have changed; a strategy employed by other researchers also working with heterogenous populations [14, 15, 17]. During the course of the SAS-CGI and CASS development, another AS-specific CGI was published [18]. The concept coverage is similar between these except that the SAS-CGI measures described here also assess seizures, cognitive impairment, and daily living/self-care issues. Additionally, expressive communication and not receptive communication was included as this was considered the most impactful aspect of communication by all the participants interviewed.

It is important to capture the perspectives of both clinicians and caregivers to understand the impact of AS symptoms and all the aspects of possible treatment-related outcomes. This is exemplified by a recently developed Duchenne muscular dystrophy measure [15]. The CASS measures described here capture a global assessment of the impact and change in AS symptoms from a caregiver’s perspective. The item wording and examples listed for each measurement domain are tailored specifically to caregivers. For instance, while the SAS-CGI-S focuses on severity of symptoms, the CASS-I focuses on the impact of symptoms. Hence, these measures are complimentary tools that could be incorporated into clinical trials to assess changes from both the clinician and caregiver perspective in response to targeted AS therapies.

Feedback from clinicians, PAs, and caregivers highlighted the importance of clearly defining the reference population when assessing AS symptom severity or impact. There is a risk of a floor effect with a reference population comprised solely of neurotypical people and there is a need to accurately capture the size of any change. Therefore, rating guidance and a scale manual were provided for the SAS-CGI to support consistent reporting, including in relation to the reference population. Similarly, the instructions in the CASS-I were clarified to direct caregivers to complete the measure comparing their child with the reference population.

The psychometric properties evaluating validity and reliability need to be further explored for these measures within clinical trial settings, including the levels of change in AS symptoms and functional impairment that would be considered meaningful by clinicians and caregivers. Importantly, interviews with clinicians and PAs suggested that the CASS measures may not be suitable for children aged < 1 year and modifications may be needed to account for the developmental expectations of this age group. Further CD research with caregivers of individuals with AS and expert clinicians is also needed to confirm the relevance of the measures for assessing adolescents older than 12 years of age and adults with AS, although the concepts included in the measures have been reported to be relevant across all ages [7].

Based on feedback from both clinicians and caregivers, improvement in expressive communication is a key meaningful change. However, further insights suggest that it may be important to consider receptive communication for future iterations of the measures. Based on the performance of the measures in clinical trials, refinement may be required, which could warrant further CD.

## Conclusions

In conclusion, we present here the development of novel AS-specific CGI measures, as well as novel AS-specific caregiver-reported measures. These both specifically and holistically assess clinician and caregiver perspectives and can be used to provide valuable insight into the severity, impact, and meaningful change in individuals with AS. Arguably, given that clinician reports of a condition can differ compared with caregivers, the assessment from both perspectives will provide a fuller picture of treatment outcomes [19].

These COAs have been incorporated into ongoing studies (NCT04428281 and NCT05100810) to assess changes in AS symptoms, which will allow further refinement based on ongoing feedback from clinicians and caregivers.

## Methods

AS-specific, clinician- and caregiver-reported COA measures for use in clinical trials were developed consistent with best practice according to FDA guidance (Fig. 1) [11, 12].

### Development of draft clinician- and caregiver-reported measures

A conceptual disease model of AS developed by Willgoss, et al. [7] plus a review of key literature were used to support the development of Global Impression measures that capture clinicians’ or caregivers’ perspective. In total, four measures were developed including two clinician-reported measures (Symptoms of AS-CGI of Severity [SAS-CGI-S] and Symptoms of AS-CGI of Change [SAS-CGI-C]) and two caregiver-reported measures (Caregiver-reported AS Scale for Impact [CASS-I] and Caregiver-reported AS Scale for Change [CASS-C]). The SAS-CGI-S and CASS-I includes a total of nine items; the SAS-CGI-C and CASS-C includes an additional item capturing the meaningfulness of change (Fig. 4) [7].

The SAS-CGI-S items each contain six response options, ranging from “None” to “Very severe” (Fig. 3), with a recall period of 2 weeks. Each of the CASS-I items contain five response options, ranging from “Not at all difficult” to “Very difficult” (Fig. 3), with a recall period of 7 days. Finally, the SAS-CGI-C and CASS-C items each contain seven response options, ranging from “Very much improved” to “Very much worse” (Fig. 3), with a recall period referring to the past 2 weeks relative to the baseline severity or since the beginning of the clinical study, respectively. To provide supporting information on the chosen severity or change level, the SAS-CGI measures also include a free text box so clinicians can note any relevant information that aided the selection of the response option. At the end of the SAS-CGI-C and CASS-C measures, the clinicians were asked to indicate if any change they had reported in the child’s overall AS in the preceeding question was meaningful or not.

### Participants and recruitment

Interviews were conducted with five clinicians with at least 5 years of experience in treating AS, five patient advocates (PAs) associated with AS-specific patient advocacy groups (PAGs), as well as caregivers (n = 15) to children with AS aged 1–12 years. Caregivers, clinicians, and PAs were independently recruited through a third-party recruitment agency. Caregivers were additionally recruited through AS-specific PAGs. The sponsor did not know the identity of any participant. Demographic data of caregivers and their children with AS, and the professional history data of clinicians and PAs were collected and summarized to characterize the total sample, and to demonstrate the robustness of the qualitative interview data. To ensure relevance to the spectrum of AS experiences, severities, and functional abilities, caregivers of children with AS were recruited for all genotypes and a broad age range (1–12 years) (Table 2).

### Interviews to discuss draft measures

Two rounds of approximately 60-min, one-to-one telephone interviews were conducted with clinicians and PAs, using semi-structured interview discussion guides. All interviews were conducted by experienced qualitative interviewers and were audio recorded, then transcribed verbatim.

#### Round 1 interviews with clinicians and PAs to discuss draft measures

Round 1 interviews consisted of a brief concept elicitation (CE) involving an open-ended discussion of the core signs, symptoms, and impacts of AS, followed by an in-depth cognitive debriefing (CD) of the initial versions of the measures. Clinicians reviewed the SAS-CGI measures, and PAs reviewed the CASS measures; participants were provided with the relevant measures prior to the interview. Given the length of this version of the measures and the 60-min interview length, not all items were debriefed with all clinicians or PAs. Some measure items were skipped to ensure feedback on all items of the measures by at least one participant.

Interview transcripts were subject to thematic analysis that involved reading the transcripts to identify codes or patterns in the data, to identify the trends in the responses, and to confirm key symptoms and impacts of AS [20]. These codes were then reviewed and merged to develop key themes that were reflective of participants’ language. The CE portion of these interviews were coded to identify which symptoms or impacts were spontaneously discussed and which were noted as being important when probed by the interviewer. Coding of CD data focused on quotes that pertained to the measures, including their relevance, comprehension, suitability of response options, any suggestions for rewording, and the measures’ coverage of important aspects of AS. To better understand how raters completing the measures would assess severity and if further clarification in the measures’ instructions were needed, the reference population that interviewees would consider to be appropriate was also discussed and analyzed. For example, it was asked whether raters would rate a given child’s severity comparing the child with the broader population of children without AS or only comparing the child with other children with AS. Additionally, the feasibility of the recall period was analyzed during Round 1 interviews. The draft versions of the SAS-CGI and CASS measures were revised based on this interview feedback, plus input from an external expert on sleep and seizures.

#### Round 2 interviews with clinicians, PAs, and caregivers to discuss revised measures

Round 2 interviews with clinicians and PAs focused on CD of the revised versions of the measures. Feedback on the following aspects were elicited: understanding of the measures’ instructions, understanding of each item and relevance to the AS patient population, suitability of the response options and recall period, and suggestions for improvements. Clinician telephone interviews for the SAS-CGI measures were extended to 90 min to ensure all aspects were debriefed and to allow further discussion about the “average” severity of AS symptoms, and what clinicians considered “normal” development among neurotypical children aged 1–12 years in order to better inform the instructions and guidance for administering the SAS-CGI measures.

For the CASS measures, PAs were asked about the relevance of the items for caregivers to individuals with AS aged 1–12 years. In-depth CD telephone interviews lasting approximately 60 min were conducted with caregivers who were sent a hardcopy of the revised CASS measures prior to the interview, but were asked not to open these in advance. A “think aloud” approach was used during the interview whereby the caregivers verbalized their initial thoughts while reading and completing the questions [21]. The interviewer actively probed with follow-up questions to explore: the participant’s understanding of the measures, the feasibility of the recall period, and how they would be able to answer if the child with AS were away (i.e., at school). Coding of CD data from the caregiver interviews focused on identifying quotes related to the interview questions and probes, similar to the approach taken in coding the CD portion of clinician and PA interviews. The response options selected by each caregiver for each item of the CASS measures were extracted from the interviews and qualitatively analyzed alongside the demographic and genetic subtype of the child with AS. This qualitative analysis was performed to identify caregiver-selected response options that did not align with expectations based on demographics and clinical characteristics of the child, in order to better understand how the measure may perform.

Final revisions were made to the SAS-CGI and CASS measures based on this feedback and a review of the extracted caregiver response data.

## Data Availability

Individual patient level data generated from this study are not publicly available; aggregated data in the form of a report may be provided by the authors upon reasonable request. Requests to access the aggregated data should be directed to the corresponding author. The current version of the SAS-CGI is distributed by MAPI Research Trust ePROVIDE™.

## Abbreviations

AS: Angelman syndrome
CASS: Caregiver-reported Angelman syndrome scale
CASS-C: Caregiver-reported Angelman syndrome scale for change
CASS-I: Caregiver-reported Angelman syndrome scale for impact
CD: Cognitive debriefing
CE: Concept elicitation
CGI: Clinician Global Impression
CGI-C: Clinician Global Impression—change
CGI-S: Clinician Global Impression—severity
COA: Clinical outcome assessment
FDA: US Food and Drug Administration
MD: Doctor of medicine
PA: Patient advocate
PAG: Patient advocacy group
SAS-CGI: Symptoms of Angelman syndrome—Clinician Global Impression
SAS-CGI-C: Symptoms of Angelman syndrome—Clinician Global Impression of Change
SAS-CGI-S: Symptoms of Angelman syndrome—Clinician Global Impression of Severity

## References

[1] Dagli AI, Mathews J, Williams CA. Angelman Syndrome. 1998 Sep 15 [Updated 2021 Apr 22]. In: Adam MP, Everman DB, Mirzaa GM, et al., editors. GeneReviews® [Internet]. Seattle (WA): University of Washington, Seattle; 1993–2022. https://www.ncbi.nlm.nih.gov/books/NBK1144/. Accessed 8 Aug 2022.

[2] Luk HM, Lo IFM (2016). Angelman syndrome in Hong Kong Chinese: a 20 years’ experience. Eur J Med Genet.

[3] Mertz LG, Christensen R, Vogel I, Hertz JM, Nielsen KB, Grønskov K, et al. Angelman syndrome in Denmark. Birth incidence, genetic findings, and age at diagnosis. Am J Med Genet A. 2013;161a:2197–203. 10.1002/ajmg.a.36058. 10.1002/ajmg.a.36058 23913711

[4] Yakoreva M, Kahre T, Žordania R, Reinson K, Teek R, Tillmann V (2019). A retrospective analysis of the prevalence of imprinting disorders in Estonia from 1998 to 2016. Eur J Hum Genet.

[5] Clayton-Smith J, Laan L (2003). Angelman syndrome: a review of the clinical and genetic aspects. J Med Genet.

[6] Grieco JC, Romero B, Flood E, Cabo R, Visootsak J (2019). A conceptual model of Angelman syndrome and review of relevant clinical outcomes assessments (COAs). Patient.

[7] Willgoss T, Cassater D, Connor S, Krishnan ML, Miller MT, Dias-Barbosa C (2021). Measuring what matters to individuals with Angelman syndrome and their families: development of a patient-centered disease concept model. Child Psychiatry Hum Dev.

[8] Wheeler AC, Sacco P, Cabo R (2017). Unmet clinical needs and burden in Angelman syndrome: a review of the literature. Orphanet J Rare Dis.

[9] Duis J, Nespeca M, Summers J, Bird L, Bindels-de Heus KG, Valstar M (2022). A multidisciplinary approach and consensus statement to establish standards of care for Angelman syndrome. Mol Genet Genomic Med.

[10] Krishnan ML, Berry-Kravis E, Capal JK, Carpenter R, Gringras P, Hipp JF (2021). Clinical trial strategies for rare neurodevelopmental disorders: challenges and opportunities. Nat Rev Drug Discov.

[11] US Food and Drug Administration. Methods to identify what is important to patients & select, develop or modify fit-for-purpose clinical outcomes assessments. In: Patient-focused drug development guidance public workshop on Guidance 2. 2018. https://www.fda.gov/media/116276/download. Accessed 8 Aug 2022.

[12] US Food and Drug Administration. Methods to identify what is important to patients & select, develop or modify fit-for-purpose clinical outcomes assessments. In: Patient-focused drug development guidance public workshop on Guidance 3. 2018. https://www.fda.gov/media/116277/download. Accessed 8 August 2022.

[13] Haro JM, Kamath SA, Ochoa S, Novick D, Rele K, Fargas A, et al. The Clinical Global Impression-Schizophrenia scale: a simple instrument to measure the diversity of symptoms present in schizophrenia. Acta Psychiatr Scand Suppl. 2003;(416):16–23. 10.1034/j.1600-0447.107.s416.5.x. 10.1034/j.1600-0447.107.s416.5.x 12755850

[14] Neul JL, Glaze DG, Percy AK, Feyma T, Beisang A, Dinh T (2015). Improving treatment trial outcomes for Rett syndrome: the development of Rett-specific anchors for the Clinical Global Impression scale. J Child Neurol.

[15] Staunton H, Trennery C, Arbuckle R, Guridi M, Zhuravleva E, Furlong P (2021). Development of a clinical global impression of change (CGI-C) and a caregiver global impression of change (CaGI-C) measure for ambulant individuals with Duchenne muscular dystrophy. Health Qual Life Outcomes.

[16] Guy W. ECDEU Assessment Manual for Psychopharmacology: U.S. Dept. of Health, Education, and Welfare, Public Health Service, Alcohol, Drug Abuse, and Mental Health Administration, National Institute of Mental Health, Psychopharmacology Research Branch, Division of Extramural Research Programs. 1986;76–338.

[17] Busner J, Targum SD. The clinical global impressions scale: applying a research tool in clinical practice. Psychiatry (Edgmont). 2007;4:28–37. PMC2880930 20526405

[18] Kolevzon A, Ventola P, Keary CJ, Heimer G, Neul JL, Adera M (2021). Development of an adapted clinical global impression scale for use in Angelman syndrome. J Neurodev Disord.

[19] Freyer DR, Lin L, Mack JW, Maurer SH, McFatrich M, Baker JN (2022). Lack of concordance in symptomatic adverse event reporting by children, clinicians, and caregivers: implications for cancer clinical trials. J Clin Oncol.

[20] Braun V, Clarke V (2006). Using thematic analysis in psychology. Qual Res Psychol.

[21] Patrick DL, Burke LB, Gwaltney CJ, Leidy NK, Martin ML, Molsen E (2011). Content validity–establishing and reporting the evidence in newly developed patient-reported outcomes (PRO) instruments for medical product evaluation: ISPOR PRO good research practices task force report: part 2–assessing respondent understanding. Value Health.

